# Long-term effectiveness and costs of a brief self-management intervention in women with pregnancy-related low back pain after delivery

**DOI:** 10.1186/1471-2393-8-19

**Published:** 2008-05-30

**Authors:** Caroline HG Bastiaenen, Rob A de Bie, Johan WS Vlaeyen, Mariëlle EJB Goossens, Pieter Leffers, Pieter MJC Wolters, Janneke M Bastiaanssen, Piet A van den Brandt, Gerard GM Essed

**Affiliations:** 1 Department of Epidemiology, Maastricht University, Maastricht, The Netherlands; 2 Department of Medical, Clinical and Experimental Psychology, Maastricht University, Maastricht, The Netherlands; 3 Department of Physiotherapy, Hogeschool Zuyd, Heerlen, The Netherlands; 4 Department of Obstetrics and Gynaecology, University Hospital Maastricht, Maastricht, The Netherlands; 5 Department of Epidemiology, Maastricht University, P.O.Box 616, 6200 MD Maastricht, The Netherlands

## Abstract

**Background:**

Pregnancy-related low back pain is considered an important health problem and potentially leads to long-lasting pain and disability. Investigators draw particular attention to biomedical factors but there is growing evidence that psychosocial and social factors might be important. It prompted us to start a large cohort study (n = 7526) during pregnancy until one year after delivery and a nested randomized controlled intervention study in the Netherlands.

**Methods:**

A randomized controlled trial (n = 126) nested within a cohort study, of brief self-management techniques versus usual care for treatment of women with persisting non-specific pregnancy-related low back pain three weeks after delivery. Women in the intervention group were referred to a participating physiotherapist. Women in the usual care group were free to choose physiotherapy, guidance by a general practitioner or no treatment. Follow up took place at 3 months, 6 months and one year after delivery.

Outcomes included change in limitations in activities (RDQ), pain (VAS), severity of main complaints (MC), global feeling of recovery (GPE), impact on participation and autonomy (IPA), pain-related fear (TSK), SF-36, EuroQol and a cost diary. For the outcome measures, series of mixed models were considered. For the outcome variable global perceived effect (GPE) a logistic regression analysis is performed.

**Results:**

Intention-to-treat outcomes showed a statistical significant better estimated regression coefficient RDQ -1.6 {-2.9;-0.5} associated with treatment, as well as better IPA subscale autonomy in self-care -1.0 {-1.9;-0.03} and TSK -2.4 {-3.8;-1.1} but were not clinical relevant over time. Average total costs in the intervention group were much lower than in usual care, primarily due to differences in utilization of sick leave but not statistically significant.

**Conclusion:**

Brief self-management techniques applied in the first 3 months after delivery may be a more viable first-line approach but further research is needed to draw inference on costs and to determine whether no care is a better option in the long term.

**Trial Registration:**

[ISRCTN08477490]

## Background

Pregnancy-related low back pain is a frustrating health problem in the Netherlands and Scandinavia because of its high prevalence during pregnancy (77–84%) [1-5] and the clinical belief that it could lead to long-lasting pain and disability after delivery [2] but prevalence drops significantly to 35% [3-5] in the first month after delivery and stabilizes directly after. Nevertheless, these figures do not gain a clear understanding into the severity of pain, limitations in activities and restrictions in participation. Researchers have been unable to identify etiologic factors [5]. We assume it is rather a subjective experience comprising pain, fatigue and a feeling of instability in the pelvic girdle and legs, starting during pregnancy. In spite of the limited knowledge of etiology, authors in this research field often hypothesized a distinction between pregnancy-related pelvic girdle and lumbar pain and based that on the assumption that pelvic girdle and lumbar pain have different etiologies [6-8], different prognoses[9] and require therefore different treatment strategies. Additionally, researchers disregard to a large extend the favorable prognosis in the first month after delivery when planning their interventions during pregnancy [10-13]. Only little scientific evaluation of treatment programs after delivery is available with just one study focusing on specific stabilizing exercises with a positive result [14]. Over the last decade we have seen a change from a biomedical approach to a biopsychosocial approach in the musculoskeletal disorders research [15] but very limited in this particular group of patients [16]. Nevertheless, there is evidence that psychosocial factors influence pain and disability and in particular the transition from acute to chronic pain [15,17].

It prompted us to start a large longitudinal, prospective cohort study (n = 7526), which studies the prevalence, etiology, severity and prognosis of pregnancy related low back and/or pelvic girdle pain during pregnancy until one year after delivery [4] and a nested randomized controlled intervention study (n = 126)[18,19].

The aim of the trial is to determine the effectiveness and cost of brief self-management techniques versus an approach based on a pain contingent basis (usual care), after delivery.

Because of the lack of a clear definition we used an extensive description of pregnancy-related low back pain including all women who experience some form of pregnancy-related pain in the lower back and/or pelvic girdle originated within the musculoskeletal system. In this study we refer to the International Classification of Functions (ICF) as a basis for describing [20] and better understanding of pregnancy-related low back pain. The ICF describes functioning and disability as an interactive process and takes a neutral stand with regard to etiology. The present article describes the longitudinal effectiveness and costs during the year after delivery. The study design [18] and the short-term results (3 months after randomization) [19] are described elsewhere.

The trial was assigned to an international trial identification number (ISRCTN08477490).

## Methods

### Recruitment and informed consent

The medical ethics committee of the Maastricht University Hospital approved the intervention and cohort study that were performed in the Southeast of the Netherlands. Midwives and gynecologists recruited the women during early pregnancy (10–14 weeks). Women were included in the cohort if they were at least 18 years old, pregnant and well versed in Dutch language. They were given written information explaining the aims and contents of the cohort and intervention study before they decide to participate. A woman entered the intervention study at the moment of three weeks after delivery after signing informed consent for both the cohort and intervention study during early pregnancy and meeting the in- and exclusion criteria of the intervention study, at three weeks after delivery. Women were included when having pain in the lower back including the pelvic girdle with an onset during pregnancy or just after delivery (cohort data), were restricted in their normal daily activities because of pregnancy-related low back pain and if there was a delay in recuperation. Women diagnosed with systematic diseases or specific pathology in the region of the pelvic girdle and/or lower back, affecting pain and activities were excluded. Exclusion also occurred in case of extensive family related or psychosocial problems or when a disablement procedure was not yet finished. Final important aspects for in-/exclusion were the willingness to participate in the study or having a clear treatment preference [21]. We only included women who did not indicate such a preference.

### Randomization and blinding

Randomization took place after collecting baseline data and obtaining informed consent. An independent research assistant unaware of the baseline data carried out the concealed randomization procedure using a computer-generated random numbers list. Block size was four. Women were told that to the current knowledge the both treatment options are considered to be equally effective. Participating physiotherapists were not involved in the baseline and effect measurements. Researchers dealing with the baseline and outcome data assessment were blinded to the intervention assignments.

### Study Interventions

All participating physiotherapists were experienced and specialized in treating women with pregnancy-related low back pain. Prior to the trial, specialized members of the Royal Dutch Physiotherapy Association, and working in the research area (Southeastern part of the Netherlands) were contacted and asked to participate in the intervention study. After signing consent they were at random but stratified according practice residence split up in two groups. The physiotherapists providing the experimental intervention received additional training and education, preceding and during the trial. The other group received no extra training (usual care group). Participating physiotherapists of both groups were asked not to communicate the contents of both treatment approaches with each other.

#### Usual care

Women, allocated to the usual care group, were free to choose usual care treatment by a physiotherapist not providing the experimental intervention, guidance by a general practitioner or do nothing. Information about the option chosen was collected by means of questionnaires during the follow-up period. When a woman chose usual care, treatment started within one week. The only interventions that were not allowed were those associated with the experimental intervention. Prior to the trial, detailed information is gathered about the contents of the current treatment options in the Netherlands. Part of the information is collected by means of group discussions with experienced physiotherapists and interviews on an individual basis with affected women out of our cohort study [4]. The program is described in detail elsewhere [18,19] and included:

• An expert role of the physiotherapist in relation to the patient focusing on disease management,

• A pain contingent regime of avoiding and limiting several specific day-to-day activities,

• Treatment goals were focused on biomedical factors,

• Stabilizing exercises of the lumbar spine and pelvic girdle.

The general practitioner in the Netherlands gives some general information about the health problem concerning the prognosis after delivery. In general, this guidance is limited to one visit.

#### Experimental intervention

Women, allocated to the experimental intervention group, were immediately referred to a participating physiotherapist in their own neighborhood. We provided an individualized self-management approach of 7–9 sessions for 30 minutes in a period of time of 12 weeks. Self-management refers to the individual's ability to manage the symptoms, treatment, physical and psychosocial consequences and life style changes inherent to living with a chronic condition [22-24]. A relationship in which the physiotherapist and the woman make health care decisions together was a basic assumption of this intervention. The program was based on brief self-management [22-24] and fear-avoidance techniques [25] and is described in detail elsewhere [18] and included:

• Standardized information by means of a protocol for the therapists and booklets for the patients specially developed for this study,

• Simple complaint-related problem-solving techniques that engaged women in identifying day-to-day problems or limitations related to the complaints under investigation,

• Setting personal goals by action planning,

• Reviewing the action plans and progress towards goals,

• A shift from an expert role of the physiotherapist to an equal partnership between physiotherapists and patients. The physiotherapist becomes a teacher in the development patient's skills to manage her health problem,

• A hierarchy of individual fear-eliciting movements and activities,

• Specific skills such as specific stabilizing exercises of the lumber spine and pelvic girdle and building up fitness training.

### Measurements

Measurement should take place in all relevant domains of the ICF; body functions, limitations in activities, participation restrictions and contextual factors. Movement related body functions are listed during physical examination. Pain, another body function is listed with two Visual Analog Scales [31,32] and with questions about pain localization and duration of pain during history taking. The level of activity is investigated with the Roland Disability Questionnaire (RDQ) [28] and the Main Complaints [29,30]. Restrictions in participation are measured with the Impact of Participation and Autonomy (IPA) [33,34]. Contextual factors include all factors that influence how disability is experienced by an individual like age, social background, profession, past and current experience. Most factors are listed during history taking and some by questionnaires during the cohort study. Personal factors are measured with the Beck Disability Inventory (BDI)[43], Pain Catastrophizing Scale (PCS)[42], Negative Emotionality Scale (NEM) [45], Positive Emotionality Scale (PEM)[45], Tampa Scale for Kinesiophobia (TSK) [35-37], Short Form-36 (SF-36) subscale "general health" [38,39], Pain Behavior Scale (PBS) [26,27] and Global Perceived Effect (GPE) [29]. Finally the EuroQol covered all domains [40].

#### History, physical examination and baseline measurement

During the home visit, a standardized history was taken and physical examination was performed by a research-physiotherapist. Demographic characteristics and data about life style, delivery including epidural anesthesia, education, medication, the onset of pain and functional status during pregnancy were already gathered as part of the cohort study. After history taking a short standardized clinical examination program was performed to exclude specific pathology, list the mobility of the back and lower extremities, observe daily activities such as walking, standing and sit down and test nerve root radiation. Because of the limited evidence, specific tests for pain provocation, pelvic stability and hyper mobility were not used as exclusion criteria or to discriminate between pelvic girdle and low back pain. The research-physiotherapist filled out the Pain Behavior Scale PBS [26,27], after the home visit.

##### Baseline measures (self-reported questionnaires)

• Beck Depression Inventory (BDI) [43], measures depressive symptoms. It is a 21-question four-point scale for measuring the severity of depression and is composed of items relating to depression such as hopelessness, irritability, cognitions as well as physical symptoms. Psychometric qualities are satisfactory and the BDI is able to discern the psychosocial from the physiological component of pain [44]. Analyses of the BDI in this study did not include items concerning weight loss, sleeping disturbance and work inhibition [44].

• Pain catastrophizing was measured with the Pain Catastrophizing Scale (PCS) [42]. The PCS consists of 13 items describing thoughts and feelings that individuals may experience when they are in pain. A 5-point scale is used with the endpoints (0) not at all and (4) all the time. The PCS has adequate psychometric qualities.

• Treatment expectancy [46] was measured by means of a 100 mm Visual Analog rating Scale (VAS). The women were asked to what extent they believed the treatment to be beneficial. There is little information regarding validity and reliability.

• To measure the experience of negative affect we used the 14-item Negative Emotionality Scale (NEM). To measure positive affect we used the 11-item Positive Emotionality Scale (PEM). Both are subscales of the Multidimensional Personality Questionnaire [45]. The tendency to experience unpleasant or negative emotional states is assessed with the NEM. One's level of pleasurable engagement with the environment was assessed with 11-item PEM. Each item is answered as true or false. Psychometric qualities are adequate.

• The Pain Behavior Scale (PBS) [26,27] is an observation scale tapping 8 pain behaviors that the physiotherapist completes after physical examination. These are verbal complaints, vocal complaints, facial grimaces, standing posture, mobility, body language, use of visible supportive equipment and stationary movement (filled out by the research physiotherapist). It is a three point scale with the endpoints, no observable pain behavior and frequent observable pain behavior. The scale is relatively independent of pain intensity and activities and psychometric qualities are adequate.

#### Outcome measures

##### Primary outcomes

• Limitations in activities were measured with the Dutch translation of the 24-item Roland Disability Questionnaire (RDQ) [28], with a higher score indicating poorer functioning. The RDQ is derived from the Sickness Impact Profile, a health status measure that covers all aspects of physical and mental function. The RDQ is specially related to physical functions that were likely to be affected by low back pain. In this study, each item was qualified with the phrase "because of my back and/or pelvic girdle". Women completing the RDQ were asked to choose between yes or no besides the statements of the questionnaire. The RDQ score is calculated by adding up the number of positive statements. The score range from 0 (no disability) to 24 (maximum disability). Psychometric qualities turned out to be very good in a wide range of populations with low back pain.

• Global Perceived Effect [29] (GPE) was measured by self-assessment on a 7-point scale (1 = completely recovered, 7 = worse than ever). The women were asked to score their perceived change three months after randomization, 6 months and 12 months after delivery. We considered "completely recovered and much improved "as a clinically important difference. There is little information regarding validity and reliability.

• The main complaints (MC) were selected by the woman herself by selecting three essential activities in a standardized way of her everyday life that at that time (baseline) difficult or impossible to perform because of low back pain and/or pelvic girdle complaints. The severity of the complaints was rated with Visual Analog rating Scales (VAS) [29,30]. For this study, only the first main complaint was used. Psychometric qualities are satisfactory [29,30].

##### Secondary outcomes

• Pain was measured with two 100-points Visual Analog rating Scales (VAS) of the McGill Pain Questionnaire (MPQ-DLV) [31,32] to record the intensity of pain during the last week and day. Endpoints of the scale are free of pain and unbearably pain. Psychometric qualities are very good.

• The impact on participation and autonomy (IPA) measured person-perceived restriction in participation and autonomy [33,34]. The IPA assesses two aspects of participation: (1) perceived participation for each item and perceived problem for each sub domain. In this study we used only the items of each sub domain. The used sub domains were self-care and appearance, mobility and leisure, social relationships and family role. Perceived participation is graded on a 5-point rating scale ranging from very good (0) to very poor (4). For each domain the participation score is calculated by summing the item scores. Higher scores denote more restrictions in participation. Psychometric qualities are satisfactory.

• Fear of movement was measured by the Dutch translation of the Tampa Scale for Kinesiophobia (TSK) [35]. The TSK is a 17-item questionnaire developed to identify fear of (re)injury due to movements or activities. Items are scored on a 4-point Likert-scale from strongly agree to strongly disagree. The total score is calculated by summing the items scores. The scale contains four reverse items (4,8, 12 an 16). We used the total TSK and the both subscales "fear avoidance" and "harm" [36,37]. Psychometric qualities are good.

• The Short-Form 36 (SF-36) [38,39] evaluated health status. The SF-36 is a generic 36-item measure. It yields an 8-scale profile of physical and mental health scores. We used only the 5-item subscale "general health", a subscale that correlates highly to the physical health construct. It is a 4-point scale with the endpoints personal health is poor and likely to get worse and personal health is excellent. For calculation of the scores prescribed norm-based algorithms were used. We used the standard (4 week) recall version. Psychometric qualities of the measure are very good en tested in a wide range of populations.

• The EuroQol (EQ) was used to obtain a self-description of mobility, self-care, used activities, pain/discomfort and anxiety/depression [40]. Each dimension comprises three levels (no problems, some/moderate problems, extreme problems). A unique EQ is defined by combining one level from each of the five dimensions. Psychometric qualities are very good in a wide range of populations.

• A cost-diary [41] was used to obtain data on physical activities, health care utilization, and days of sick leave. The diary is presented in booklet form, containing instructions, and an accompanying letter explaining the objective of the diary. We asked the patients to record only disease specific resource. Women were instructed to record costs on a weekly basis until one year after delivery. Psychometric qualities are satisfactory.

#### Follow-up

Women were asked to complete follow-up questionnaires at 3 months after randomization (short-term results), 6 months and one year after delivery. The follow-up questionnaires contained the listed outcome measures. Furthermore, items on contents, satisfaction and beneficial aspects of the (experimental) treatment were listed. Co-interventions, medication, aids, additional medical consumption, recurrence of complaints, return to gainful employment and a possible subsequent pregnancy were also registered. Besides, the follow-up questionnaires assessed also how many treatment sessions were followed in the previous period of time.

Women who did not return their follow-up questionnaires were contacted by (e) – mail or phone and were asked to continue participation.

### Statistical analyses

The baseline status of the study groups was compared with respect to the distribution of the baseline values of all measures. For the outcome measures, series of mixed models were considered [47]. The models were fitted using the linear mixed function in SPSS 12.0[48]. For the categorical outcome variable GPE a logistic regression was performed. A cut-off point for distinguishing between improved and not improved was chosen. Fully recovered and much improved were considered to be improved. All analyses were intention to treat (ITT) [49]. For all women, pre-randomization values of outcomes and covariates were complete. Any response values of a woman are included in the analyses. No attempt was made to replace missing response values with imputed values. A two-tailed p-value of 0.05 was considered to indicate statistical significance. The times after baseline (in months), group, potential confounders and baseline measurement of the scale under investigation were treated as fixed effects. Multivariable analyses were performed to examine the effects for potential confounding factors measured at baseline in addition to time after baseline, baseline measurement of the scale and groups. Potential confounding factors were age, education, profession, treatment expectancy, PBS, BDI, PCS, NEM and PEM. Epidural anesthesia during delivery is an also sometimes mentioned confounding factor of importance. However, opposite to many other countries epidural anesthesia during delivery is not common practice in the Netherlands. Figures about this variable were collected but only 11 women received epidural anesthesia (6 in the control group and 5 in the experimental intervention group), so this variable was not entered in the model. Normality of the residuals was examined using normal probability plots and histograms. Subgroup analyses were performed for the subgroups baselines scores RDQ < 13 and ≥ 13 (median split) to explore a possible different intervention effect in subgroups of women with severe limitations in activities at baseline. Prognostic status at baseline for women with and without missing values for the outcome variables at 3 and 6 months and one year after delivery were compared between both groups.

A clinical important change on the primary outcome RDQ was considered a change of 2 points.

Power calculations showed that to detect a difference of 2 in changes scores of the RDQ between both groups at 80% power and with alpha = 0.05 a total sample size of 124 is needed. When alpha = 0.1 with 80% power a total sample size of 90 is needed.

#### Economic evaluation

An economic evaluation compared the costs and health effects of both treatment options from a societal perspective. Resources recorded in the cost diaries were valued by cost estimates for the year 2003. True cost estimates are available in the Dutch guidelines for pharmaco-economic studies (43, 44). Costs for over the counter drugs (OTC) and aids were reported directly by the women in their diaries. Productivity costs resulting from loss of paid labor were calculated by applying the friction costs method, which limits the period of production loss to the time during the work of the person is not replaced. In 2003 the friction period was set at 22 weeks or 154 days. In this study, the regular period of maternity leave was excluded from the friction period. The length of maternity leave after delivery is set at 10 weeks in the Netherlands. Paid production was valued by the average national gross wage per hour, broken down by sex (female) and age category (25–44 year).

Between-group differences in outcomes of mean total costs were analyzed by Student's *t*-tests for unpaired observations. Total costs are considered the primary total cost outcome.

## Results

### Recruitment

**Figure 1:**
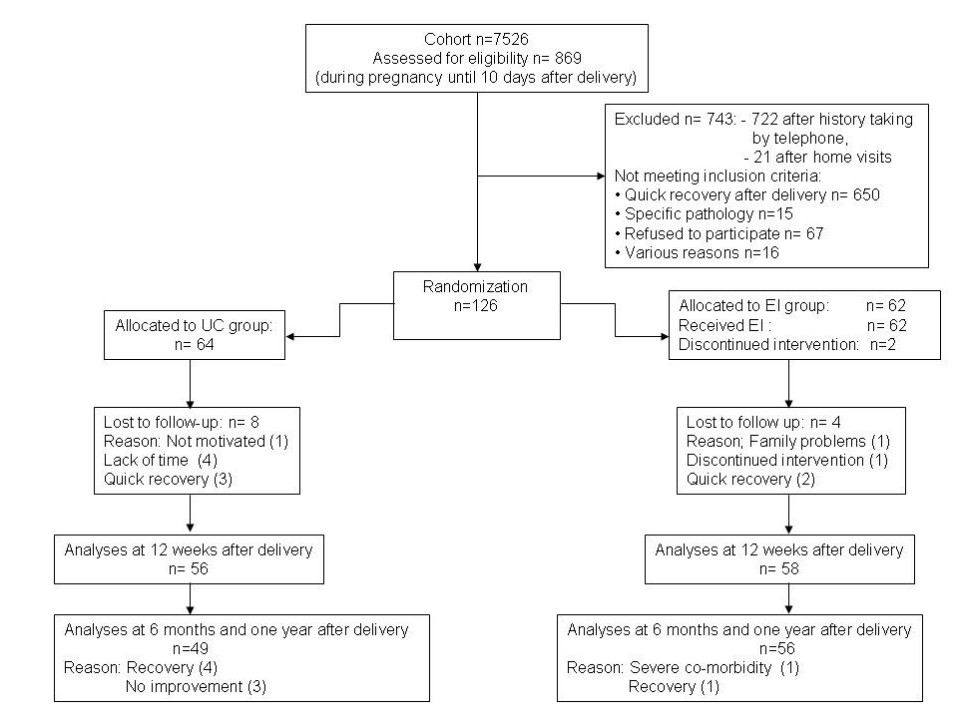
Flow participants through the study

Self-administrated questions about limitations in activities and the perceived need for treatment among women from the cohort study (n = 7526) resulted in 869 possibly eligible participants, in the first week after delivery. On basis of history taking by telephone two weeks after delivery, 743 women were excluded from participation (Figure 1). The majority of them were excluded because of a spontaneous remission within the first two weeks after delivery (n = 650). History taking by telephone resulted in 147 home visits about three weeks after delivery. Based on these home visits, 21 women were excluded. Finally, from May 2001 until July 2003, 126 women were included in the intervention study three weeks after delivery.

### Description of the study population

#### • Baseline Characteristics

Baseline status of the participants is given in Table 1. Both groups were highly similar in prognostic variables and baseline values of outcome measures.

**Table 1:** Baseline characteristics and measures according to treatment group

Variable	Usual Care (n = 64) Mean (SD)	Experimental Intervention (n = 62) Mean (SD)
Age	31.5 (3.1)	31.4 (3.6)
Localisation of pain: (n/%)		
*Lumbar spine*	25 (39.1)	24 (38.7)
*SI joints (one or two)*	34 (53.1)	34 (54.8)
*Symphysis*	43 (67.2)	42 (67.7)
History of low back pain (n/%)	43 (67.2)	44 (71.0)
Parity	2.1 (1.0)	2.3 (1.0)
***Primary outcomes***		
**VAS (pain today) **(0–100)	54.3 (17.5)	53.5 (19.1)
**VAS (pain this week) **(0–100)	59.2 (17.9)	57.0 (17.8)
**RDQ **(0–24)	13.5 (4.3)	13.3 (4.6)
***Secondary outcomes***		
**IPA**:		
*Autonomy in self-care (0–28)*	9.6 (5.7)	8.8 (5.1)
*Mobility and leisure (0–20)*	10.9 (3.7)	10.0 (4.7)
*Family role (0–28)*	15.4 (5.8)	14.4 (5.6)
*Social Relationships (0–24)*	5.8 (3.3)	5.5 (3.4)
**MC **(VAS)* (0–100)	69.5 (17.5)	72.0 (15.7)
**TSK:**		
Total score (17–68)	35.7 (5.9)	32.9 (5.0)
*Fear Avoidance† (8–32)*	18.8 (3.6)	17.0 (3.0)
*Harm ‡ (5–20)*	8.5 (2.2)	7.9 (2.3)
**PCS**	12.2 (9.8)	11.2 (8.4)
**BDI **¶	5.3 (4.9)	5.1 (4.2)
**NEM**	3.3 (3.4)	2.4 (2.3)
**PEM**	7.8 (2.6)	8.5 (2.4)
**SF-36**:		
*General Health*	54.6 (12.5)	57.8 (13.1)
Expectancy treatment	79.0 (17.8)	78.3 (16.6)
PBS # (0–8)	1.3 (1.3)	1.5 (1.6)

* first mean complaint was selected, † TSK subscale fear-avoidance (items 1,2,9,10,13,14,15,17), ‡ TSK subscale harm (items 3,5,6,7,11), ¶mean BDI calculated without items 15,16, 19, # Pain Behavior Scale filled out by research-physiotherapist after physical examination.

SI = Sacroiliac, VAS = Visual Analog rating Scale, RDQ = Roland Disability Questionnaire, IPA = Impact on Participation and Autonomy, MC = Main Complaint, TSK = Tampa Scale for Kinesiophobia, PCS = Pain Catastrophying Scale, BDI = Beck Disability Inventory, NEM = Negative Emotionally Scale, PEM = positive Emotionally Scale, SF-36 = Short-Form-36, PBS = Pain Behavior Scale

#### • Follow-up

After randomization, 64 women were assigned to the usual care group and 62 to the experimental intervention group. For 114 women (90%) data were available for all outcome measures 3 months after randomization (Figure 1). In 94% of the women of the experimental group records showed that they had received the techniques defined a priori as relevant to this intervention. Not one adverse event of the experimental intervention was recorded.

In the usual care group 42 women received treatment from a physiotherapist and 3 women received guidance from a general practitioner, in the first three months, 19 women preferred the no treatment option in that period.

In 105 women (83%) all outcome measures were complete at six months and one year after delivery. The prognostic status at baseline of women who were lost to follow-up and women who filled out all the outcome measures was highly similar.

#### • Co-intervention, recurrence of pain, return to gainful employment and a possible subsequent pregnancy

Nineteen women of the usual care group did not receive any treatment in the year after delivery. Nineteen women of the same group received treatment in the follow-up period (between three months after randomization and one year after delivery). Four women in the experimental intervention group received treatment in the follow-period.

In both groups a considerable number of women reported pain flare-ups after they became free of any pain. (usual care n = 18, experimental group n = 15). 34 women of the usual care group and 45 of the experimental group reported return to gainful employment one year after delivery. 11 women of the usual care group and 5 of the experimental group applied for a benefit due to the disorder. Four women of the usual group and six of the experimental group did not return to gainful employment because they were out of a job. Six women (three in both study groups) reported a subsequent pregnancy one year after delivery.

### Effectiveness of the experimental intervention (EI) compared to the usual care option (UC)

The outcomes of both study groups until one year after delivery are shown in table 2 and the results of the main linear effects models after the experimental intervention compared to usual care fitted to each outcome variable in table 3. The estimated treatment assignment effect demonstrates a statistical significant effect in the RDQ, for the experimental group compared to usual care. However there does not appear to be a clinically important difference in change in advantage of the experimental intervention over time (one year delivery). Adjustment for multiple comparison (Bonferronni correction) alpha would have been .016; the estimated treatment effect is still statistical significant (table 3) but the clinical relevance did not changed. Results of binary logistic regression with the dichotome outcome variable GPE did not show statistical significant difference between the both groups at 3, 6 and 12 months. The BDI is a significant co-variable at three months after delivery (p = .018); 73.7% is correctly classified. At 6 months 79.8% is correctly classified and at 12 months 84.5%. There were no significant co-variables.

**Table 2:** Outcomes of both study groups until one year after delivery

	Usual Care (3 months) N = 56 Mean (SD)	Experimental Intervention (3 months) N = 58 Mean (SD)	Usual Care (6 months) N = 49 Mean (SD)	Experimental Intervention (6 months) N = 56 Mean (SD)	Usual Care (One Year) N = 49 Mean (SD)	Experimental Intervention (One year) N = 56 Mean (SD)
***Primary outcomes***						
**RDQ **(0–24)	6.8 (5.5)	4.5 (4.9)	4.9 (4.8)	4.5 (5.4)	3.4 (4.2)	3.5 (4.9)
**MC **(VAS) (0–100)	26.8 (21.0)	21.0 (21.9)	20.5 (21.8)	19.7 (21.5)	17.8 (18.8)	17.6 (20.5)
GPE*: n/%	37 (66.1)	43 (74.1)	37 (75.5)	46 (82.1)	41 (83.6)	46 (82.1)
**GPE **p-value◇	0.28		0.47		0.5	
**VAS **(0–100) (pain today)	26.3 (19.9)	24.3 (24.1)	20.7 (20.6)	22.5 (23.5)	17.4 (19.9)	20.7 (23.1)
**VAS **(0–100) (pain this week)	29.6 (21.7)	25.3 (23.0)	22.3 (21.5)	23.2 (23.6)	18.5 (19.7)	23.7 (23.8)
***Secondary outcomes***						
**IPA**:						
*Autonomy in self-care (0–28)*	4.0 (4.6)	3.0 (4.3)	3.3 (4.2)	2.4 (4.1)	2.8 (3.5)	2.6 (4.0)
*Mobility and leisure (0–20)*	6.3 (4.0)	5.6 (4.4)	5.2 (3.8)	4.8 (4.4)	4.5 (3.4)	4.5 (4.6)
*Family role (0–28)*	9.2 (5.1)	7.8 (5.8)	7.5 (5.3)	6.6 (5.8)	5.8 (5.0)	6.0 (6.3)
*Social relationships (0–24)*	4.4 (2.9)	4.0 (3.3)	3.8 (2.9)	3.8 (3.5)	3.7 (2.8)	3.7 (3.2)
**TSK:**						
Total score (17–68)	32.4 (5.6)	28.6 (6.0)	32.3 (5.7)	28.6 (5.9)	31.0 (6.3)	28.7 (6.7)
*Activity Avoidance (8–32)*	16.2 (3.7)	13.5 (4.0)	15.7 (4.0)	13.3 (3.9)	14.8 (4.0)	13.3 (3.8)
*Harm (5–20)*	7.9 (2.4)	7.2 (2.3)	7.9 (2.6)	7.3 (2.4)	7.6 (2.5)	7.6 (2.4)
**SF-36**:						
*General Health (0–100*)	59.2 (13.7)	61.4 (14.0)	60.9 (13.8)	61.9 (13.0)	62.4 (13.9)	61.2 (16.2)
**EuroQol **(0–1)	0.7 (0.2)	0.7 (0.2)	0.7 (0.2)	0.7 (0.2)	0.79 (0.1)	0.75 (0.2)

Ratings on a 7-point scale are dichotomized as improved (completely recovered and much improved) and not-improved (slightly improved, not changed and slightly/much/vastly worsened).

◇ results of binary logistic regression; p-value

RDQ = Roland Disability Questionnaire, MC = Main Complaint, VAS = Visual Analog rating Scale, IPA = Impact on Participation and Autonomy, TSK = Tampa Scale for Kinesiophobia, Fear Avoidance = subscale TSK consisting items 1,2, 9, 10, 13, 14, 15, 17, Harm = subscale TSK consisting items 3, 5, 6, 11, SF 36 = Short Form 36

**Table 3:** Longitudinal analyses for main linear mixed models (n-114). Parameter estimates and CI intervals for treatment effects in outcomes, after experimental intervention compared to usual care

Outcome measure	Estimated regression coefficient {95% CI } P-value (two-tailed)
***Primary outcomes***	
RDQ (0–24)	-1.6 {-2.9;-0.5} 0.005
MC (VAS) * (0–100)	-4.9 {-10.3;0.4} 0.07
Secondary outcomes	
VAS (pain today) (0–100)	-1.4 {-6.6;3.8} 0.58
VAS (pain this week) (0–100)	-3.6 {-9.3;2.0} 0.20
***Secondary outcomes***	
IPA:	
*Autonomy in self-care (0–28)*	-1.0 {-1.9;-0.03} 0.04
*Mobility and leisure (0–20)*	-0.4 {-1.3;0.5} 0.40
*Family role (0–28)*	-0.7 {-1.9;0.5} 0.27
*Social relationships (0–24)*	-0.3 {-0.8;0.2} 0.23
TSK:	
Total score (17–68)	-2.4 {-3.8;-1.1} 0.00
*Activity Avoidance (8–32)*^‡^	-1.7 { -2.6;-0.8} 0.00
*Harm (5–20)*^†^	-0.3 {-0.8;0.2} 0.23
SF-36:	
*General Health (0–100)*	-0.7 {-3.3;1.8} 0.57
EuroQol (0–1)	-0.005 {-0.04;0.03} 0.87
**Subgroup RDQ < 13**	
TSK (total) (17–68)	-1,9 {-3.6;-0.15} 0.03
*Activity Avoidance (8–32)(TSK-subscale)*	-1.3 {-2.3;-0.3} 0.02
**Subgroup RDQ ≥ 13**	
RDQ (0–24)	-1.8 {-3.4:-0.14} 0.03
*Autonomy in self-care (0–28)(IPA subscale)*	-1.7 {-3.0;-0.4} 0.01
*Activity Avoidance (8–32)(TSK subscale)*	-1.5 {-2.7;-0.3} 0.01

First main complaint was selected, ^‡ ^TSK subscale Activity Avoidance (items 1,2, 9,10, 13, 14, 15, 17),

^† ^TSK subscale Harm (items 3, 5, 6, 7, 11).

RDQ = Roland Disability Questionnaire, MC = Main Complaint, VAS = Visual Analog rating Scale, IPA = Impact on Participation and Autonomy, TSK = Tama Scale for Kinesiophobia, SF-36 = Short Form-36.

Secondary significant estimated treatment assignment effects are demonstrated in the total score of the TSK and subscale Activity Avoidance and the IPA subscale Autonomy in self-care. The estimated treatment effect in the IPA subscale Autonomy in self-care also demonstrates a significant effect for the experimental group compared to usual care. Depression measured with the BDI was a significant covariate in all models.

Other estimated treatment assignment effects were not significant different although there was an extensive within-subject improvement on the primary outcome RDQ (about 10 points improvement on the RDQ from baseline to one year after delivery) in both study groups and on several secondary outcomes (Tables 1, 2). Subgroup analyses showed that results were in line with the results of the main analyses. The subgroup with baseline values on the RDQ ≥ 13 showed a significantly greater reduction on the same outcomes in favor of the experimental intervention (RDQ, TSK, Activity Avoidance and Autonomy in self-care) as the main analyses (Table 3). Additionally, results of the subgroup RDQ < 13 showed a significantly greater reduction in the Activity Avoidance scale and the total score of the TSK, after experimental group compared with usual care.

Women in both groups reported a substantial reduction in pain (VAS pain today and last week) in the year after delivery (Table 2), but there were no significant differences between either study groups or subgroups (Table 3).

Histograms of the changes scores of the RDQ suggested that the assumption of normal distributed scores was acceptable (range between -5 and 20). Floor or ceiling effects are considered to be present if more than 15% of the respondents achieved the lowest or highest possible score. At baseline, only one participant showed the highest possible score (24) on the RDQ and no one the lowest possible score (range 23). With a mean of 13.4, SD 4.4 for the total group and inspection of the histogram, the normal distribution of the baseline variable was also acceptable. There were no floor or ceiling effects of the RDQ to be present at baseline. Histograms of the residuals of the linear mixed models suggested that the assumptions of normality distributed variables were acceptable.

### Costs of the experimental intervention compared to the usual care option

The sessions of the experimental intervention (7–9) were included in the total amount of physiotherapists' sessions. Costs were listed in table 4. Differences between the mean total costs were in favor of the experimental group. Although not significant, the huge differences between the groups were almost entirely due to the differences in costs of sick leave after finishing maternity leave. Costs of sick leave were about double in the usual care group compared to the experimental group. Mean total costs of the subgroups baseline RDQ score < 13 and ≥ 13 were in line with the costs of the total study groups. Results were confirmed in the subgroup analyses. The majority of the direct costs (more than the costs of the experimental intervention (€ 210,-)) were generated by 67.5% of the women in the usual care group and 33.9% of the experimental intervention group.

**Table 4:** Mean (SD), total costs (€) and differences between both study groups

	Experimental intervention N = 56 Mean SD	Usual Care N = 49 Mean SD	Differences between groups Mean 95%CI
Direct Costs	288.7 (248.1)	354.3 (336.9)	-65.6 {-180;49}
Indirect costs (without sick leave)	388.6 (864.4)	470.2 (741.8)	-81.6 {-397.7;234}
Indirect costs (including sick leave)	3601.6 (8576)	7689.3 (12012.7)	-4087.7 {-8501;326.4}
Total costs (without sick leave)	671.2 (978)	7689.3 (12012.7	-4087.7 {-8501;326.4}
Total costs (including sick leave)	3862.6 (8700)	8203.7 (12229.5)	-4341.1 {-8850;167.7}
**Subgroup baseline score RDQ • 13**	N = 32	N = 27	
Total costs (without sick leave)	814.2 (1219.2)	1113.3 (1097.1)	-299.1 {-920.1;322}
Total costs (including sick leave)	3902.3 (8887)	8628 (12451.4)	-4725.6 {-11082.7;1631}
**Subgroup baseline score RDQ < 13**	N = 24	N = 22	
Total costs (without sick leave)	486.6 (493.6)	489.4 (649.4)	-2.84 {-347.2;341.5}
Toatal costs (including sick leave)	3815.4 (8690.6)	7734.8 (12302)	-3919.4 {-10686;1006.3}

Calculation of cost-effectiveness ratios was not relevant because both the effectiveness of the primary and secondary outcomes and reduction in costs were in favor of the experimental intervention.

## Discussion

### Key findings

In spite of the statistical significant estimated treatment effect of the RDQ for the experimental group compared to the usual care group, the difference is too small to be clinically relevant over time (one year after delivery). Nevertheless, subgroup analyses indicated that women with more severe limitations in activities benefited more from the experimental intervention compared to women in the usual care group on the primary outcome RDQ.

In spite of the large confidence intervals of costs due to sick leave within both study groups, the mean costs of sick leave in the usual care group were about doubled compared to the experimental intervention group; however not statistically significant. Differences remained stable in the subgroup analyses. Maybe, the experimental intervention has some influence on sick leave. Physiotherapists of the experimental intervention were instructed in detail about the advice return to work. They were not allowed to advice against return to work but were asked to encourage the women in their intention to return to work with the support of goal-orientated action plans. In the usual care group, return to work was a regular topic of conversation but left to the appraisal of the physiotherapist to advice a woman. At the same time, there were still many other unknown reasons why a considerable number of the young mothers were reluctant to return to gainful employment after finishing maternity leave. More research in this field in general and especially in the Dutch situation is necessary to investigate potential prognostic variables that influence the duration of sick leave related to pregnancy and childbirth. We did not perform mixed models in this evaluation because we had not enough information about probably influential confounders. Results of the economic evaluation in this trial were therefore of limited significance.

Recurrence of pain episodes was a quite common phenomenon in the year after delivery but seems to be independent from the differences in improvement on resumption of normal activities, participation (work) and reduced fears between the both study groups. Pain flare-ups seemed to be better manageable in the experimental intervention group than in the usual care group.

In-/exclusion data show extensive improvement in the first weeks after delivery preceding the enrollment in the intervention study. The improvement within both groups lasted until one year after delivery. These results indicate that pregnancy-related low back pain is a temporary albeit inconvenient condition with a good prognosis, especially in the first month after delivery.

Overall, results of this trial are for the greater part in line with trials using self-management approach with chronic low back pain patients (16, 45).

#### Experimental intervention

Participating therapists were already embedded in the primary health care system. Both the educational course received by the therapists and the intervention they delivered were brief and appropriate for implementation in primary care. Therapists showed the potential to shift the model of care from a biomedical approach to a broad approach that incorporated psychosocial factors. The shift from a biomedical to a biopsychosoial approach is not to mistake or misunderstand. A biopsychosocial attitude is not a matter of "leaving or ignoring the biomedical domain" but a matter of integrating all relevant aspects and paying attention to diagnostics as well as intervention regarding impairments, activities and participation. Collaborative partnership and shared responsibility between physiotherapists and the women become important. Action planning and problem solving need training and evaluation, education alone is not enough. An example of shared responsibility is that signs and symptoms must not be ignored but physiotherapists and women have to learn to interpret then adequately. Either important is to learn which activities are helpful to become active again. Graded exposure of the activities that were avoided was integrated in the protocol. After an initial period of learning these skills, it becomes the task of the woman themselves in collaborative partnership with their physiotherapist. For both the women and the physiotherapists it was a rather new policy.

#### Limitations of the study

Similarity of improvement between the both study groups at one year after delivery raises the question whether either approach is superior to the other or to no treatment at all. Unfortunately, it was not possible to design a study with a third group receiving no treatment after delivery because there was a strong urge to leave open the possibility of a referral to a physiotherapist already in the first month after delivery. The urge did not only come from the potential participants but also in an even greater extend from the physiotherapists. All included women in the study wanted to be referred to a physiotherapist at the moment of enrolment in the intervention study. Nevertheless, a considerable number of women of the usual care group did not visit a physiotherapist afterwards. This was quite unforeseeable and has not only influenced the primary outcome but also the huge variation in costs in the usual care group.

A restriction on our study and on future studies is that the prevalence of the disorder after delivery is low. We used the maximum of participants out of the cohort (n = 7526) and still could include only 126 women. This could have influenced the power of the study.

#### Strengths of the study

The trial had high internal validity shown by an adequate recruitment out of the cohort, remote system of randomization, blinding of the assessors and researchers involved in the measurements and analyses. Embedding the trial in a large cohort study had the advantage that it enabled us to specify more precisely not only the participants but also an optimal time-frame for the intervention. According to the prevalence figures of pain during pregnancy and after delivery, the enrolment data out of the cohort and the follow-up data of the intervention study, the transition from acute to chronic pain falls most likely within the timeframe of the first two or three months after delivery. This is a moment of special interest for more detailed diagnostics and intervention. Start of the experimental intervention shortly after delivery was reasonable well-timed.

#### Research and clinical implications

Several authors in this research field advocate an intervention study during pregnancy (11–13) with a follow-up in the first year after delivery. The high prevalence of pain during pregnancy seems to support this opinion. However, the moment of transition from acute to chronic pain seems to lie in the first two months after delivery. Second, the huge amounts of participants that will be needed to reach sufficient power speak against an intervention study during pregnancy.

The findings of this study and the underpinning results of the subgroup analyses lead to a future study question including a brief self-management approach compared to a no intervention and a usual care option. A study like this will address the effectiveness of the self-management approach and cost in more highly selected groups with greater disability. Potential confounders related to return to work should be included too. The preferable time-frame of the experimental intervention is between 3–4 weeks until three months after delivery.

At least six out of every seven women with pain during pregnancy make a rapid recovery in the first two weeks after delivery. Nevertheless, every woman that is seeking for help during pregnancy must be offered some form of simple guidance, in clinical practice. A stay active approach and information about the prevalence and natural course of the disorder seems to be worthwhile aspects.

An important signal to actually start with an active intervention after delivery in the future is not only pain and limitations in activities but also women's own worries and needs. Evaluation of the complaints within the framework of the biopsychosocial approach turned out to be meaningful.

## Conclusion

Brief self-management techniques applied during the first 3 months after delivery are probably a viable first-line approach for the management of pregnancy-related low back pain, but further research is needed to get more insight into the interference of the costs and a comparison with a no treatment option. Secondary, results indicate that it is a temporary disorder with a good functional prognosis especially in the first months after delivery.

## Competing interests

The authors declare that they have no competing interests.

## Authors' contributions

All authors read and approved the final manuscript. CHGB, RAdB, JWSV, MEJBG, PMJCW, JMB, PAvdB, GGME: Study concept and design. CHGB, RAdB, PL, JMB: Analysis and interpretation of the data. CHGB, RAdB:Drafting the manuscript. RAdB, JWSV, MEJBG, PL, JMB, PAvdB, GGME: Critical revision of the manuscript for important intellectual content. CHGB, RAdB, JMB: Statistical Analysis.

## Pre-publication history

The pre-publication history for this paper can be accessed here:


